# Supplementary material for: Collateral activity of the CRISPR/RfxCas13d system in human cells
Source: Commun Biol. 2023 Mar 28;6:334. doi: 10.1038/s42003-023-04708-2 (PMC10049998; doi:10.1038/s42003-023-04708-2)
Supplement: Supplementary file 2 — Supplementary information [file 42003_2023_4708_MOESM2_ESM.pdf]

Supplementary Information for

Collateral activity of the CRISPR/RfxCas13d system in human cells

Peiguo Shi[1,*], Michael R. Murphy[1], Alexis O. Aparicio[1], Jordan S. Kesner[1], Zhou Fang[1], Ziheng Chen[1,2], Aditi Trehan[1], Yang Guo[1], Xuebing Wu[1,*]

[1]Department of Medicine and Department of Systems Biology, Columbia University Irving Medical Center, New York, NY 10032, USA

[2]Current address: Department of Biological Sciences, Carnegie Mellon University, Pittsburgh, PA 15213, USA

*Correspondence to: ps3008@cumc.columbia.edu (P.S.), xw2629@cumc.columbia.edu (X.W.)

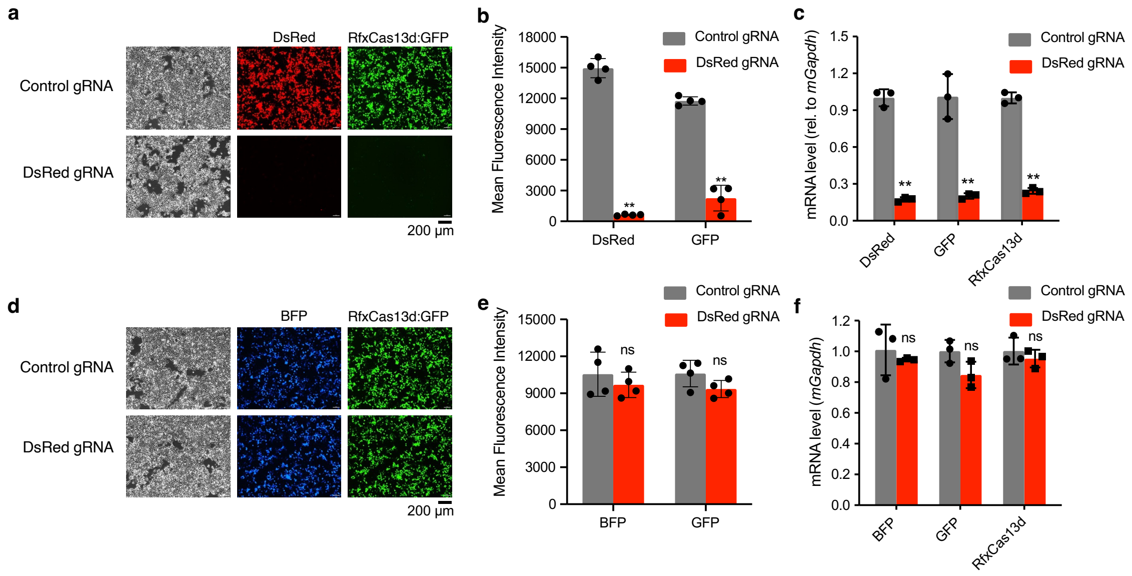

Supplementary Figure 1. The loss of BFP/GFP depends on the target of the gRNA (i.e., DsRed). (**a-c**), Cells were transfected with RfxCas13d-2A-GFP, DsRed, and either a DsRed targeting gRNA or a non-targeting control gRNA. The fluorescent images (**a**) were quantified in (**b**) and the mRNA changes were quantified in (**c**). (**d-f**), same as (**a-c**) but replacing DsRed with BFP. Note that BFP/GFP are only down-regulated when DsRed is present and targeted by RfxCas13d loaded with a DsRed gRNA. Two-tailed Student's *t*-test was used. **: $p<0.01$. N=3-4 for all panels. Scale bar, 200 µm. Error bars represent standard deviation.

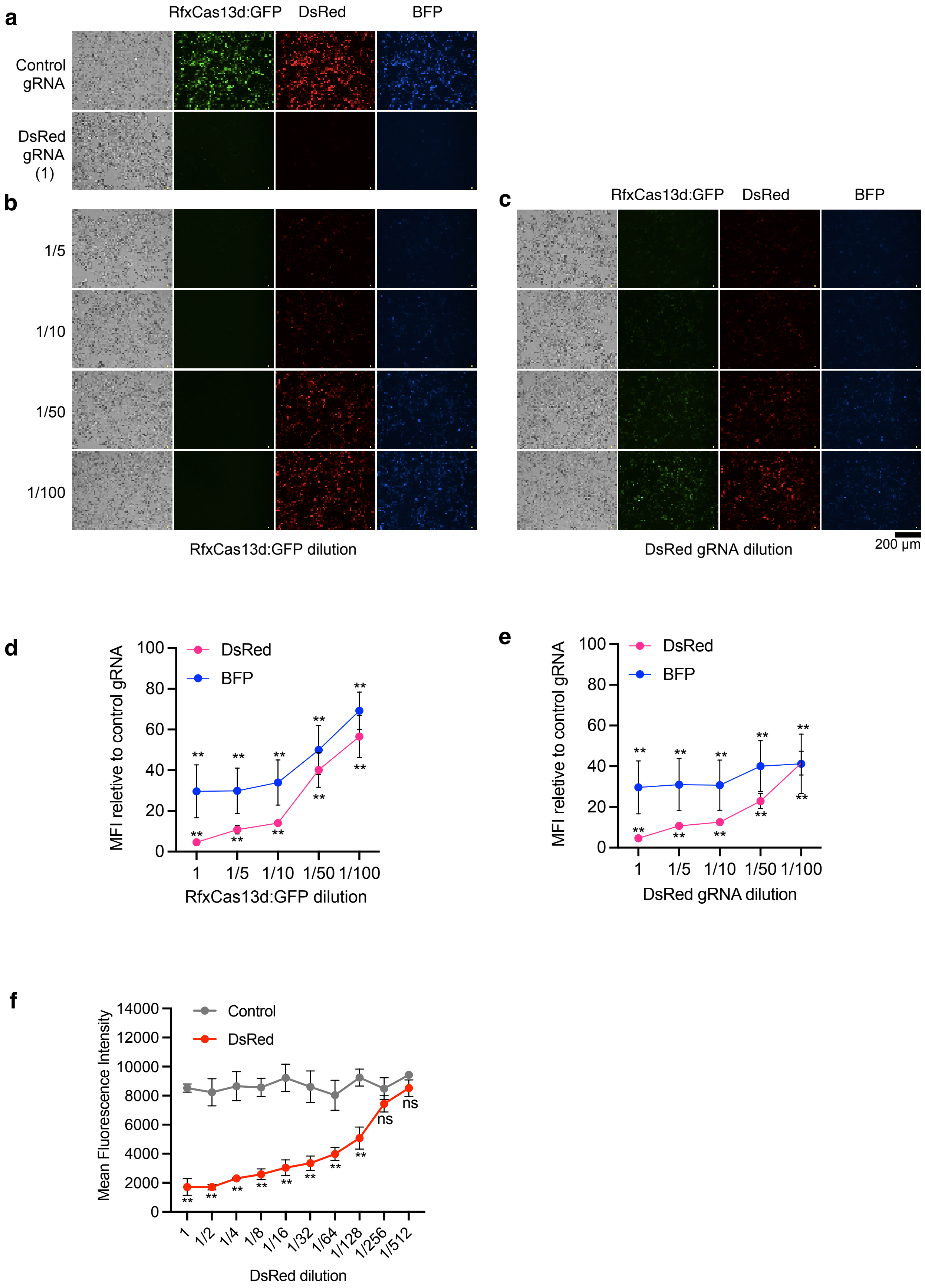
a
RfxCas13d:GFP
DsRed
BFP
Control gRNA
DsRed gRNA (1)
b
1/5
1/10
1/50
1/100
RfxCas13d:GFP dilution
c
RfxCas13d:GFP
DsRed
BFP
DsRed gRNA dilution
200 μm
d
MFI releative to control gRNA
DsRed
BFP
1
1/5
1/10
1/50
1/100
RfxCas13d:GFP dilution
e
MFI releative to control gRNA
DsRed
BFP
1
1/5
1/10
1/50
1/100
DsRed gRNA dilution
f
Mean Fluorescence Intensity
Control
DsRed
ns
1
1/2
1/4
1/8
1/16
1/32
1/64
1/128
1/256
1/512
DsRed dilution

Supplementary Figure 2. The effect of titrating Cas13d and gRNA expression on collateral activity and on-target knockdown efficiency. (**a**) Fluorescent imaging of cells transfected with Cas13:GFP, DsRed (target), BFP, and either a control gRNA (top row) or a DsRed gRNA (bottom row). (**b**) same as (**a**) (bottom) except Cas13:GFP expression is reduced by transfecting 5, 10, 50, or 100-fold less plasmids. (**c**) Similar to (**b**) but DsRed gRNA expression is reduced. (**d-e**), quantification of (**b-c**), respectively; DsRed and BFP compared with the control group DsRed and BFP, respectively. (**f**) Quantification of BFP signal in **Fig. 1i**., which was then used to generate **Fig. 1j**. Two-way ANOVA was used. **: $p<0.01$. N=3 for all panels. Scale bar, 200 μm. Error bars represent standard deviation.

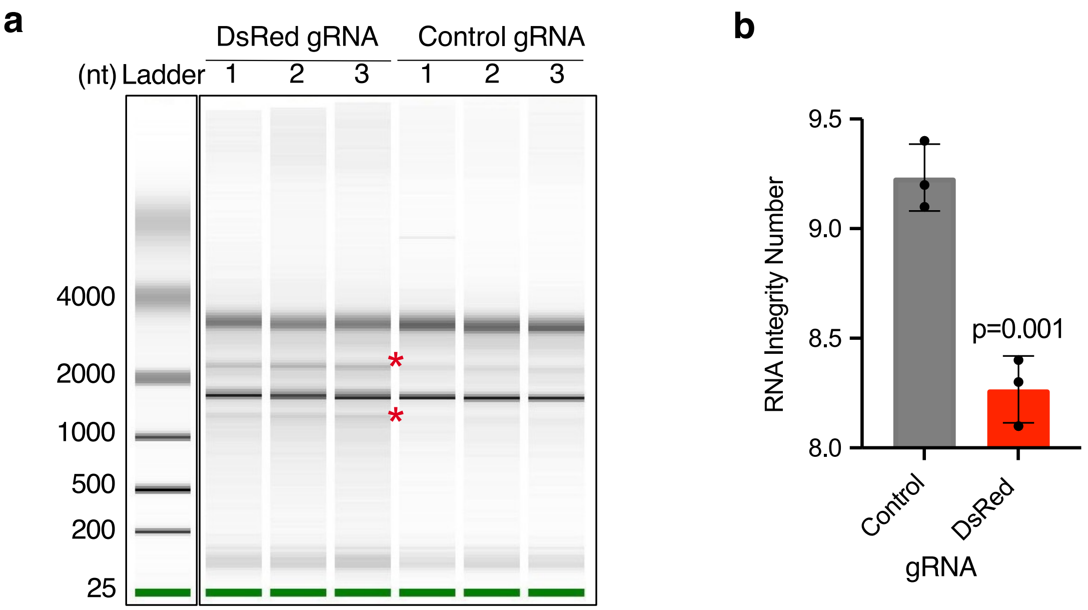

Supplementary Figure 3. Targeting DsRed with CRISPR/RfxCas13d led to rRNA degradation (**a**, BioAnalyzer) and a significant reduction of RNA integrity number (RIN) (**b**). The asterisks indicate potential degradation products of the rRNA. Note that the absolute decrease of the RIN number (~10%) is smaller than the decrease in total RNA content (**Fig. 1f**, ~46%), presumably because RIN measures RNA fragmentation and does not capture fragmented RNAs that have been degraded completely by cells prior to RNA extraction. P value was calculated using two-tailed Student's *t*-test. N=3. Error bars represent standard deviation.

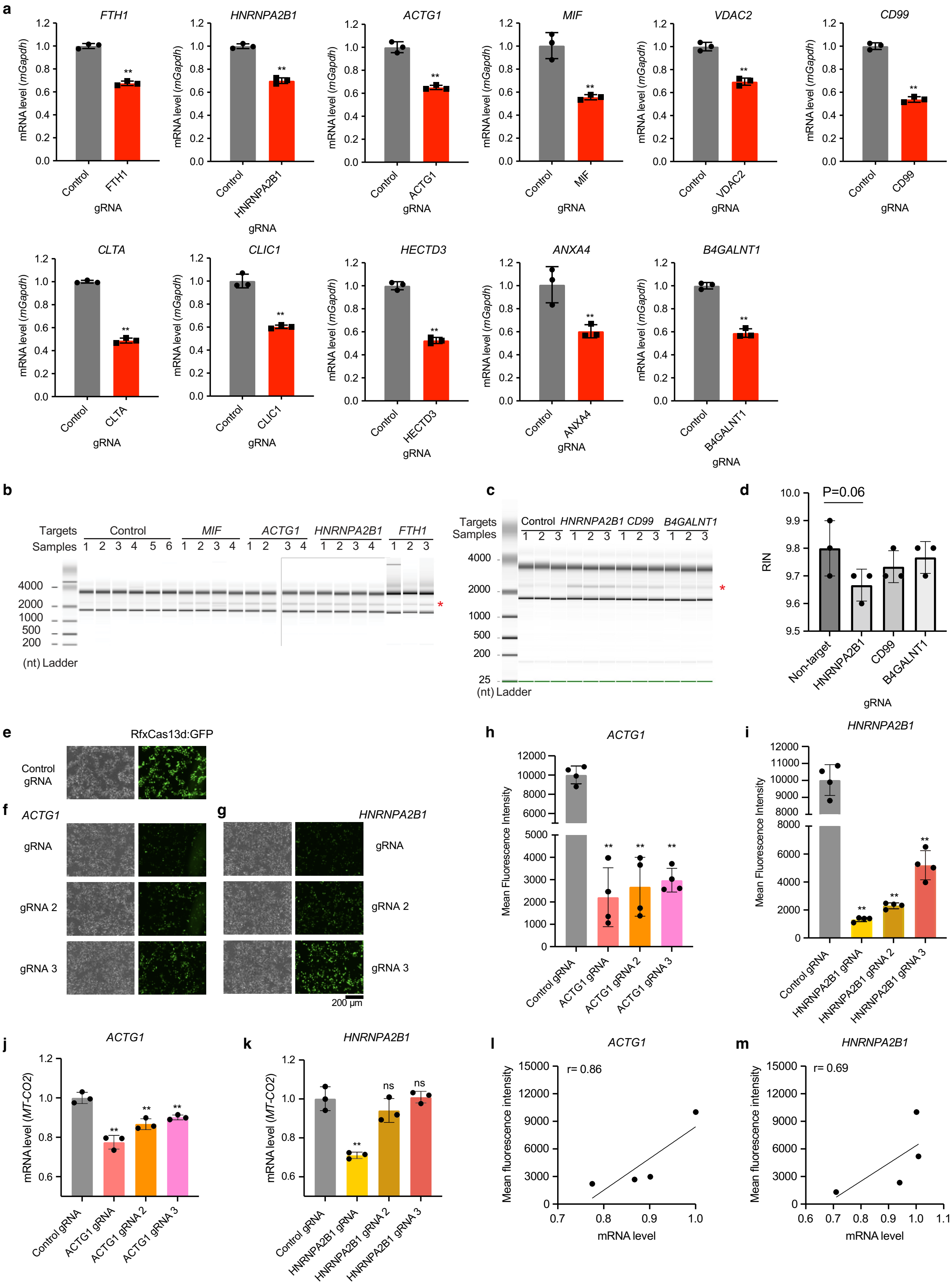
a
FTH1
HNRNPA2B1
ACTG1
MIF
VDAC2
CD99
CLTA
CLIC1
HECTD3
ANXA4
B4GALNT1
mRNA level (mGapdh)
Control
gRNA
**
b
Targets
Samples
Control
MIF
ACTG1
HNRNPA2B1
FTH1
4000
2000
1000
500
200
(nt) Ladder
*
c
Targets
Samples
Control
HNRNPA2B1
CD99
B4GALNT1
4000
2000
1000
500
200
25
(nt) Ladder
d
P=0.06
RIN
Non-target
HNRNPA2B1
CD99
B4GALNT1
gRNA
e
RfxCas13d:GFP
Control gRNA
f
ACTG1
gRNA
gRNA 2
gRNA 3
g
HNRNPA2B1
200 μm
h
ACTG1
Mean Fluorescence Intensity
Control gRNA
ACTG1 gRNA
ACTG1 gRNA 2
ACTG1 gRNA 3
i
HNRNPA2B1
Mean Fluorescence Intensity
Control gRNA
HNRNPA2B1 gRNA
HNRNPA2B1 gRNA 2
HNRNPA2B1 gRNA 3
j
ACTG1
mRNA level (MT-CO2)
k
HNRNPA2B1
mRNA level (MT-CO2)
ns
l
ACTG1
r= 0.86
Mean fluorescence intensity
mRNA level
m
HNRNPA2B1
r= 0.69
Mean fluorescence intensity
mRNA level

Supplementary Figure 4: RfxCas13d-mediated knockdown of endogenous targets. (**a**) qPCR quantification of knockdown efficiency for each target. (**b**) Targeting four abundant endogenous transcripts resulted in rRNA degradation (BioAnalyzer assay similar to **Supplementary Fig. 3**). Three to six biological replicates were shown for each target. (**c**) Similar to (**b**) but comparing high, medium, and low abundance targets. (**d**), RNA integrity number (RIN) quantified from (**c**). (**e-m**) Cells transfected with RfxCas13d:GFP, and a control gRNA (**e**), ACTG1 gRNAs (**f**), or HNRNPA2B1 gRNAs (**g**). The fluorescent images (**e-g**) were quantified in (**h-i**) and the mRNA changes were quantified in (**j-k**), respectively. The correlation between target mRNA knockdown and collateral activity (GFP) for *ACTG1* and *HNRNPA2B1* targeting were shown in (**l-m**), respectively. r: Pearson's correlation coefficient. Two-tailed Student's *t*-test for (**a**). Two-way ANOVA test for (**d**, **h-k**). **: $p<0.01$. N=3 or 4 for all panels. Error bars represent standard deviation.

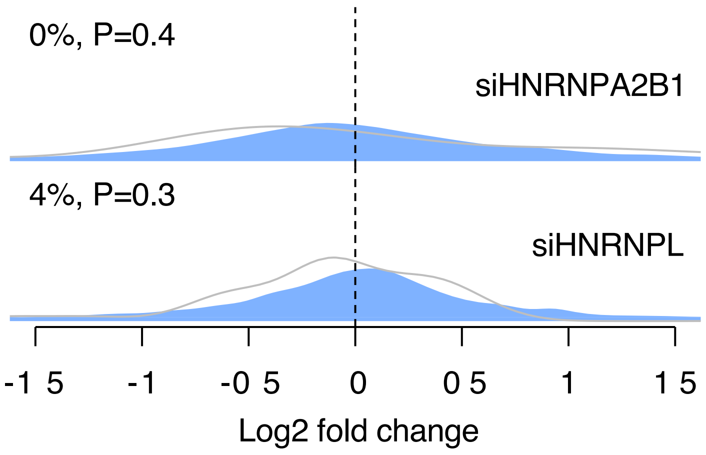

Supplementary Figure 5. siRNA-mediated knockdown of *HNRNPA2B1* or *HNRNPL* did not result in down-regulation of the transcriptome (relative to mitochondrial RNA). P value was calculated using Wilcoxon Rank Sum Test.

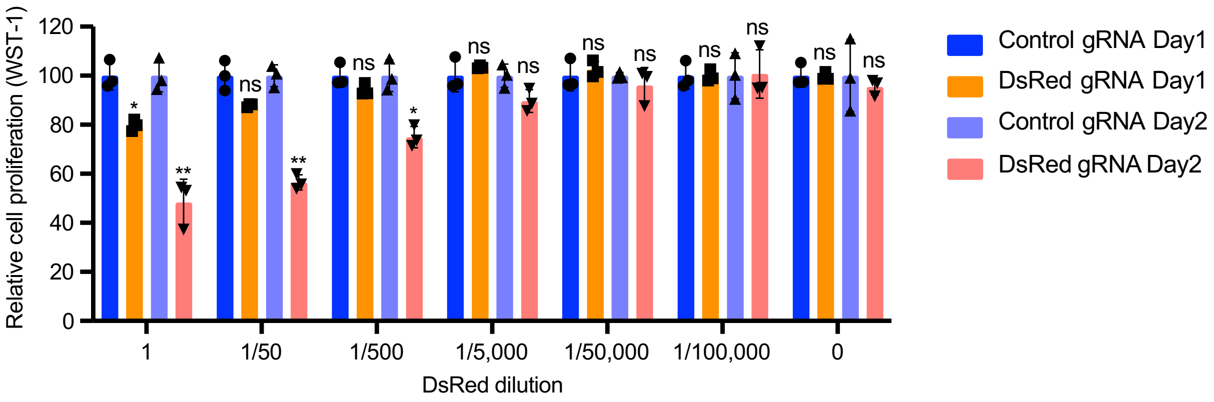

Supplementary Figure 6. Change in cell proliferation (WST-1 assay) in cells transfected with varying amount of DsRed plasmids, together with a fixed amount of RfxCas13d and gRNA plasmids. Two-way ANOVA was performed to obtain the P value. *: $p<0.05$; **: $p<0.01$. N=3. Error bars represent standard deviation.

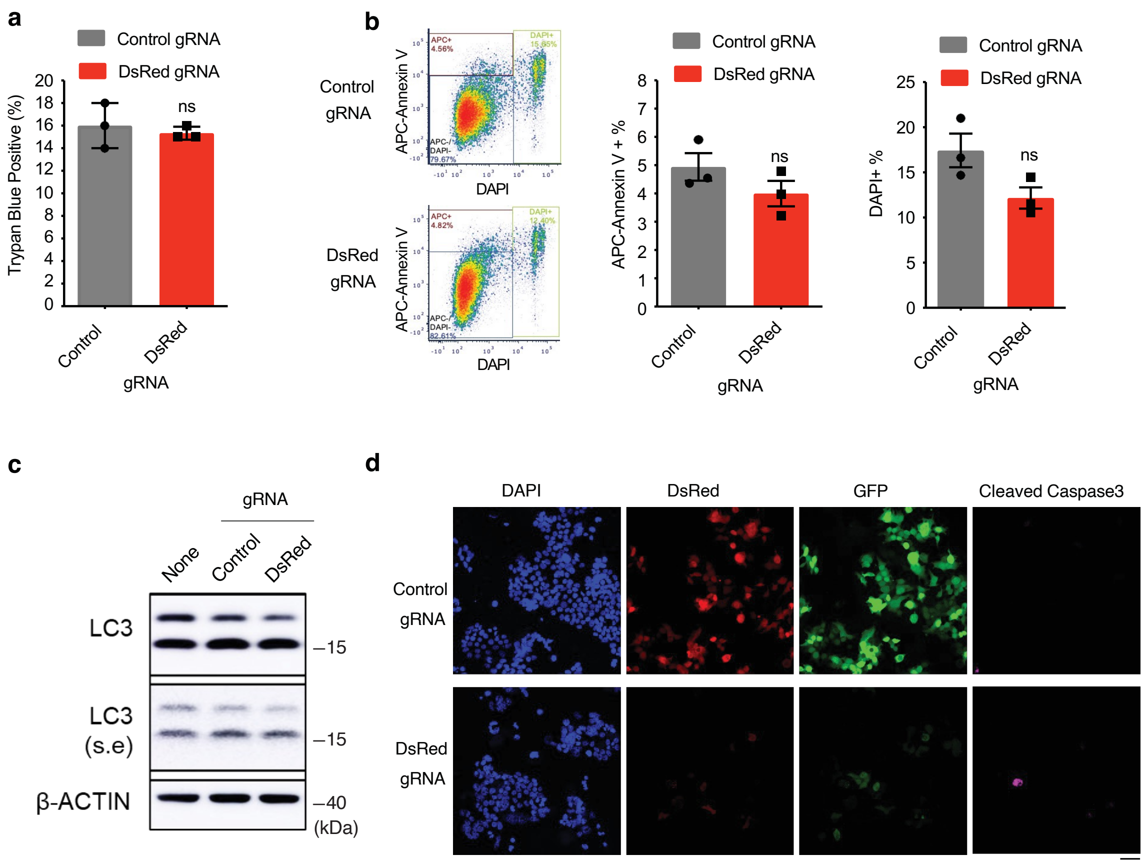

Supplementary Figure 7. DsRed-induced RfxCas13d collateral activity does not cause cell death. HEK293T cells were transfected with DsRed, RfxCas13d-2A-GFP, and either a DsRed-targeting gRNA or a non-targeting control gRNA. No difference was detected with trypan blue-based cell death assay (**a**), Annexin V - APC / DAPI cell apoptosis assay (**b**), LC3 cleavage (autophagy) (**c**), or Caspase3 cleavage (apoptosis) (**d**). Two-tailed Student's *t*-test was used to calculate P values. N=3 for all panels. Scale bar, 50 μm. Error bars represent standard deviation.

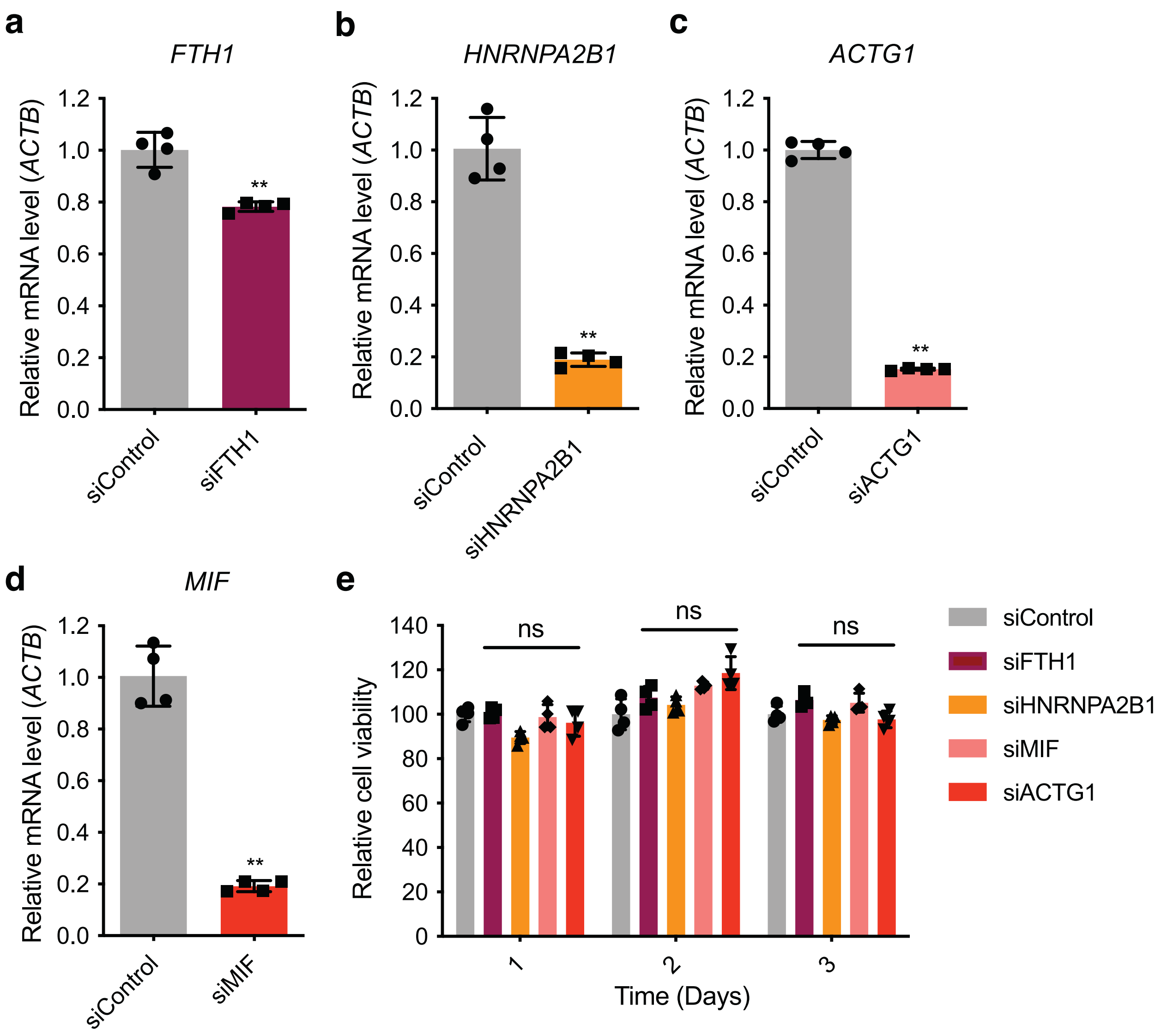

Supplementary Figure 8. siRNA-mediated knockdown of abundant endogenous mRNAs did not affect cell proliferation. (**a-d**), knockdown efficiency for *FTH1*, *HNRNPA2B1*, *ACTG1*, and *MIF*, respectively. (**e**), Relative cell proliferation (WST-1) comparing knockdown and controls. For (**a-d**), two-tailed Student's *t*-test was performed to calculate P values. For (**e**), two-way ANOVA was performed to calculate the P value. **: $p<0.01$. N=3-4 for all panels. Error bars represent standard deviation.

a

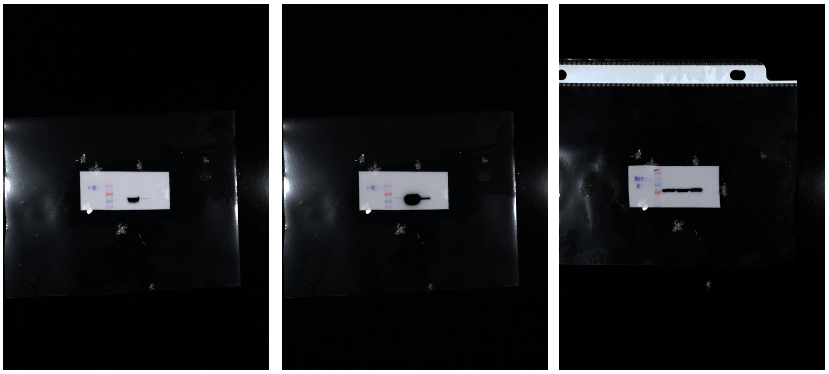

b

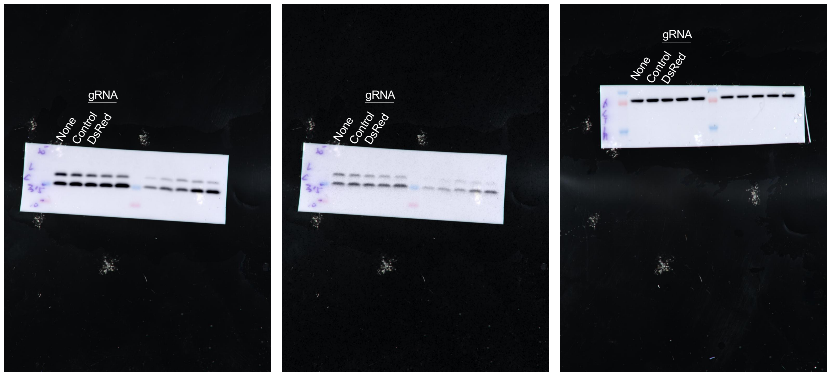

Supplementary Figure 9. (**a**) Raw gel image for (**Fig. 1d**). (**b**) Raw gel image for (**Supplementary Figure 7c**).

Supplementary Table 1: Guide RNA (gRNA) spacer sequences.

gRNA	Sequence
DsRed	GGAGGTCAAAACAGCGTGGATG
DsRed gRNA 2	TGGCGTCTCCAGGCGATCTGAC
DsRed gRNA 3	ACAGCGTGGATGGCGTCTCCAG
DsRed gRNA 4	TGTCTTCTATGGAGGTCAAAAC
DsRed gRNA 5	AGGCCGCCGGTGTCTTCTATGG
DsRed gRNA 6	CGGTAGCGCTAGGCCGCCGGTG
DsRed gRNA 7	GATCTGAGTCCGGTAGCGCTAG
DsRed gRNA 8	GCGACCGGTGGATCTGAGTCCG
DsRed gRNA 9	GGCCATGGTGGCGACCGGTGGA
DsRed gRNA 10	CCTCGGAGGAGGCCATGGTGGC
Control (for DsRed)	GTTTTGACCTCCATAGAAGACA
MIF	GTTTACGATGAACATCGGCATGA
ACTG1	AGAATGAATACATTTACAGGCGT
ACTG1 gRNA 2	CTCAAAGCAAGTAACAGCCCACG
ACTG1 gRNA 3	AGCCATTGTCAATGACCAGCGCG
HNRNPA2B1	GTTTCAAAGCTTAAGCCACCAAT
HNRNPA2B1 gRNA 2	TCCTATTTATACAGTGAAGCCCA
HNRNPA2B1 gRNA 3	CCATAGTTGTCATAACCACCTCC
FTH1	TGTCAAAGAGATATTCCGCCAAG
CLIC1	ATTTGGCAAATATGTCCAGCCCA
VDAC2	GACGTTGAAAATTCCACGCCACT
CD99	GTTAGTAAACATCATCCGCCCAG
CLTA	CAGAATAATGCACAGAAGCACAG
ANXA4	CTTTACTATAGCCAGCAGAGCAT
B4GALNT1	ATGACAAGAAGAAGCCAGTCTGC
HECTD3	TCCCAGAAATACTGCACCCGCGA
Control (for endogenous genes)	TCACCAGAAGCGTACCATACTCA

Supplementary Table 2: RT-qPCR primers sequences.

**Primer**	**Sequence**
mGapdh-qPCR-F	TGAAGCAGGCATCTGAGGG
mGapdh-qPCR-R	CGAAGGTGGAAGAGTGGGAG
MT-CO2-qPCR-F	CCGTCTGAACTATCCTGCCC
MT-CO2-qPCR-R	GAGGGATCGTTGACCTCGTC
Cas13d-qPCR-F	GGTCTACACCATGATGGACTTT
Cas13d-qPCR-R	CGCTCAGGGACTTCTCATTATC
BFP-qPCR-F	GAGAGTCACCACATACGAAGAC
BFP-qPCR-R	CCCTCTGATCTTGACGTTGTAG
GFP-qPCR-F	GAACCGCATCGAGCTGAA
GFP-qPCR-R	TGCTTGTCGGCCATGATATAG
DsRed-qPCR-F	CCCCGTAATGCAGAAGAAGA
DsRed-qPCR-R	GGTGATGTCCAGCTTGGAGT
FTH1-qPCR-F	CCCCCATTTGTGTGACTTCAT
FTH1-qPCR-R	GCCCGAGGCTTAGCTTTCATT
HNRNPA2B1-qPCR-F	TTTGGGGATGGCTATAATGG
HNRNPA2B1-qPCR-R	CTGGTTGCCATATCCAGGTC
ACTG1-qPCR-F	CCATCATGAAGTGTGACGTG
ACTG1-qPCR-R	ACACCGAGTACTTGCGCTCT
MIF-qPCR-F	TCATCGTAAACACCAACGTG
MIF-qPCR-R	GAAGGCCATGAGCTGGTC
VDAC2-qPCR-F	TTGATACTACCTTCTCACCAAACAC
VDAC2-qPCR-R	TCAAAGTCAACATCACAACCAA
CD99-qPCR-F	AACCCACCCAAACCGATGC
CD99-qPCR-R	TGAAAAGCTACCGGAGGAACTA
CLTA-qPCR-F	CTTCGCTGACGTGATTGGTTA
CLTA-qPCR-R	GCCTGTTCTAGGCTGTAGCAA
CLIC1-qPCR-F	ACCGCAGGTCGAATTGTTC
CLIC1-qPCR-R	ACGGTGGTAACATTGAAGGTG
HECTD3-qPCR-F	CATCGCCTGGGATCGAGAC
HECTD3-qPCR-R	CGCACTCGTAGGTCCATGTC
ANXA4-qPCR-F	GGAGGTACTGTCAAAGCTGCT
ANXA4-qPCR-R	GGCAAGGACGCTAATAATGGC
B4GALNT1-qPCR-F	CAGAAACAAGTCCGAGCTATTGA
B4GALNT1-qPCR-R	GAGGGGCTGAACTTCCACAC
