# Supplementary material for: Collateral activity of the CRISPR/RfxCas13d system in human cells
Source: Commun Biol. 2023 Mar 28;6:334. doi: 10.1038/s42003-023-04708-2 (PMC10049998; doi:10.1038/s42003-023-04708-2)
Supplement: Supplementary file 5 — Reporting Summary [file 42003_2023_4708_MOESM5_ESM.pdf]

Corresponding author(s): Peiguo Shi, Xuebing Wu

Last updated by author(s): Feb 20, 2023

# Reporting Summary

Nature Portfolio wishes to improve the reproducibility of the work that we publish. This form provides structure for consistency and transparency in reporting. For further information on Nature Portfolio policies, see our Editorial Policies and the Editorial Policy Checklist.

## Statistics

For all statistical analyses, confirm that the following items are present in the figure legend, table legend, main text, or Methods section.

| n/a | Confirmed | |
|---|---|---|
| ☐ | ☒ | The exact sample size ($n$) for each experimental group/condition, given as a discrete number and unit of measurement |
| ☐ | ☒ | A statement on whether measurements were taken from distinct samples or whether the same sample was measured repeatedly |
| ☐ | ☒ | The statistical test(s) used AND whether they are one- or two-sided<br>*Only common tests should be described solely by name; describe more complex techniques in the Methods section.* |
| ☒ | ☐ | A description of all covariates tested |
| ☒ | ☐ | A description of any assumptions or corrections, such as tests of normality and adjustment for multiple comparisons |
| ☐ | ☒ | A full description of the statistical parameters including central tendency (e.g. means) or other basic estimates (e.g. regression coefficient) AND variation (e.g. standard deviation) or associated estimates of uncertainty (e.g. confidence intervals) |
| ☐ | ☒ | For null hypothesis testing, the test statistic (e.g. $F$, $t$, $r$) with confidence intervals, effect sizes, degrees of freedom and $P$ value noted<br>*Give P values as exact values whenever suitable.* |
| ☒ | ☐ | For Bayesian analysis, information on the choice of priors and Markov chain Monte Carlo settings |
| ☒ | ☐ | For hierarchical and complex designs, identification of the appropriate level for tests and full reporting of outcomes |
| ☒ | ☐ | Estimates of effect sizes (e.g. Cohen's $d$, Pearson's $r$), indicating how they were calculated |

*Our web collection on statistics for biologists contains articles on many of the points above.*

## Software and code

Policy information about availability of computer code

| | |
|---|---|
| Data collection | No software was used. |
| Data analysis | GraphPad Prism 9, ImageJ, FCS Express 7, Microsoft Excel. |

For manuscripts utilizing custom algorithms or software that are central to the research but not yet described in published literature, software must be made available to editors and reviewers. We strongly encourage code deposition in a community repository (e.g. GitHub). See the Nature Portfolio guidelines for submitting code & software for further information.

## Data

Policy information about availability of data

All manuscripts must include a data availability statement. This statement should provide the following information, where applicable:

- Accession codes, unique identifiers, or web links for publicly available datasets
- A description of any restrictions on data availability
- For clinical datasets or third party data, please ensure that the statement adheres to our policy

RNA-seq data were deposited in Gene Expression Omnibus (GEO) with the accession number GSE155134.

## Human research participants

Policy information about studies involving human research participants and Sex and Gender in Research.

| | |
|---|---|
| Reporting on sex and gender | N/A |
| Population characteristics | N/A |
| Recruitment | N/A |
| Ethics oversight | N/A |

Note that full information on the approval of the study protocol must also be provided in the manuscript.

# Field-specific reporting

Please select the one below that is the best fit for your research. If you are not sure, read the appropriate sections before making your selection.

☒ Life sciences ☐ Behavioural & social sciences ☐ Ecological, evolutionary & environmental sciences

For a reference copy of the document with all sections, see nature.com/documents/nr-reporting-summary-flat.pdf

# Life sciences study design

All studies must disclose on these points even when the disclosure is negative.

| | |
|---|---|
| Sample size | No statistical methods were used to predetermine sample size. Most experiments were measuring average behavior of large number of molecules and cells, no animal was used, typically 3-4 replicates were used. |
| Data exclusions | Excluded a data point if its value is clearly an outlier compared to other replicates from the same sample, and investigators were blinded to hypothesis. |
| Replication | Most experiments were repeat at least 3 times to ensure reproducibility. |
| Randomization | The same cell line was used in all experiments when comparing treatments. |
| Blinding | Investigators were blinded during data collection and analysis by labeling samples with numbers. |

# Reporting for specific materials, systems and methods

We require information from authors about some types of materials, experimental systems and methods used in many studies. Here, indicate whether each material, system or method listed is relevant to your study. If you are not sure if a list item applies to your research, read the appropriate section before selecting a response.

### Materials & experimental systems

| n/a | Involved in the study | |
|---|---|---|
| ☐ | ☒ | Antibodies |
| ☐ | ☒ | Eukaryotic cell lines |
| ☒ | ☐ | Palaeontology and archaeology |
| ☒ | ☐ | Animals and other organisms |
| ☒ | ☐ | Clinical data |
| ☒ | ☐ | Dual use research of concern |

### Methods

| n/a | Involved in the study | |
|---|---|---|
| ☒ | ☐ | ChIP-seq |
| ☐ | ☒ | Flow cytometry |
| ☒ | ☐ | MRI-based neuroimaging |

## Antibodies

| | |
|---|---|
| Antibodies used | Cleaved Caspase 3 staining assays: Cleaved Caspase 3 antibody (Cell signaling technology, 9661T, 1:500).<br>Western blot: HA (Sigma, H9658, 1:1000), β-Actin (Sigma, A5441, 1:2000), LC3 (Proteintech, 14600-1-AP, 1:1000). |
| Validation | HA antibody was validated by using cells with and without expression of HA -tagged protein.<br>Other antibodies were validated by vendor:<br>https://www.sigmaaldrich.com/US/en/product/sigma/a5441 |

https://www.cellsignal.com/products/primary-antibodies/cleaved-caspase-3-asp175-antibody/9661
https://www.ptglab.com/products/MAP1LC3B-Antibody-14600-1-AP.htm

## Eukaryotic cell lines

Policy information about cell lines and Sex and Gender in Research

| | |
|---|---|
| Cell line source(s) | HEK293T was purchased from ATCC. MEF-1, and U87 cell lines were gifts from Muredach P. Reilly, Peter A. Sims. respectively. |
| Authentication | HEK293T was purchased from ATCC. MEF-1 and U87 cell lines were not authenticated by us. |
| Mycoplasma contamination | All cells were routinely tested for mycoplasma contamination using MycoAlertTM Mycoplasma Detection Kit (Lonza, LT07-418). |
| Commonly misidentified lines (See ICLAC register) | None |

## Flow Cytometry

### Plots

Confirm that:

☒ The axis labels state the marker and fluorochrome used (e.g. CD4-FITC).

☒ The axis scales are clearly visible. Include numbers along axes only for bottom left plot of group (a 'group' is an analysis of identical markers).

☒ All plots are contour plots with outliers or pseudocolor plots.

☒ A numerical value for number of cells or percentage (with statistics) is provided.

### Methodology

| | |
|---|---|
| Sample preparation | For apoptosis assays: Annexin V and DAPI staining assays were performed using the apoptosis detection Kit (BioLegend, 640930) according to the manufacturer's protocol. Briefly, 48 hours after transfection, cells were washed twice with cold cell staining buffer (Biolegend, 420201) and resuspended in binding buffer. APC-Annexin V and DAPI (Alternative for 7-AAD of the kit) were used for staining. Cells were incubated for 15 min at room temperature and analyzed by flow cytometry.<br><br>For competitive growth assay: A human U87 glioblastoma cell line stably expressing RfxCas13d (no GFP fusion) was generated using lentiviral integration followed by puromycin selection. Two cell lines were subsequently derived using lentiviral integration of either GFP or BFP followed by FACS. In the absence of treatment, the two cell lines, termed U87GFP cells and U87BFP cells respectively, have similar doubling time. The U87GFP cell line additionally expresses the DsRed reporter. The two cell lines were mixed at 1:1 ratio on day 0, then an in vitro transcribed gRNA targeting DsRed was transfected using Lipofectamine 3000 according to the manufacturer's protocol. Mock transfection and transfection of a non-targeting gRNA were used as control. The fraction of U87GFP and U87BFP cells were determined using flow cytometry by detecting the GFP and BFP expression. |
| Instrument | BD LSR II |
| Software | FCS Express 7 was used to analyze data. |
| Cell population abundance | At least 10,000 events were collected. |
| Gating strategy | Cells were gated based on FSC-A and SSC-A. Singlets were gated based on FSC-A and FSC-H. Positive cells were gated based on the unstained samples or untransfected parental cells. |

☒ Tick this box to confirm that a figure exemplifying the gating strategy is provided in the Supplementary Information.
